# Polymer-Metal Nanoparticle Hybrid Systems for Targeted Breast Cancer Therapy: Advances and Clinical Translation

**DOI:** 10.7759/cureus.113813

**Published:** 2026-08-02

**Authors:** Pranoti A Mane, Amar Mohite, Kailas D Datkhile, Girish R Pathade, Rahul M Sonavale

**Affiliations:** 1 Department of Biotechnology, Krishna Institute of Science and Technology, Krishna Vishwa Vidyapeeth (Deemed to be University), Karad, IND; 2 Department of Microbiology, Krishna Institute of Science and Technology, Krishna Vishwa Vidyapeeth (Deemed to be University), Karad, IND

**Keywords:** breast cancer, clinical translation, drug resistance, nanomedicine, polymer-metal hybrid nanoparticles, targeted drug delivery, theranostics, tumor microenvironment

## Abstract

Breast cancer remains a challenging malignancy in modern oncology among women worldwide and a leading cause of cancer-related mortality. Despite advances in chemotherapy, endocrine therapy, targeted therapeutics, and immunotherapy, treatment outcomes remain limited by systemic toxicity, poor tumor selectivity, multidrug resistance, and disease recurrence. Nanotechnology-based drug delivery systems have emerged as promising approaches to overcome these limitations. Among them, polymer-metal hybrid nanoparticles combine the biocompatibility and controlled drug-release properties of polymeric carriers with the unique optical, photothermal, magnetic, catalytic, and imaging capabilities of metallic nanoparticles. This narrative review highlights the synergistic advantages of polymer-metal hybrid nanoparticles and their emerging role in targeted breast cancer therapy. Recent advances in their design, synthesis, surface functionalization, targeting strategies, and multifunctional applications have expanded their potential for targeted drug delivery, photothermal therapy, and theranostic interventions. These hybrid nanoplatforms facilitate passive and active tumor targeting, support stimuli-responsive drug release, and enable integrated diagnostic and therapeutic functions. Particular emphasis is placed on their ability to modulate the tumor microenvironment and overcome multidrug resistance in clinically relevant breast cancer subtypes, including human epidermal growth factor receptor 2 (HER2)-positive, hormone receptor-positive, and triple-negative breast cancers. Preclinical studies demonstrate enhanced tumor accumulation, improved antitumor efficacy, reduced off-target toxicity, and superior imaging performance compared with conventional therapies. Despite these advances, challenges remain in large-scale manufacturing, reproducibility, long-term safety evaluation, pharmacokinetic variability, and regulatory approval. Overall, polymer-metal hybrid nanoparticles represent a versatile and promising nanomedicine platform for precision breast cancer management, with continued advances in nanoparticle engineering, safety assessment, and clinical validation expected to facilitate their successful translation into personalized oncology.

## Introduction and background

Breast cancer overview

For many years, in most countries, female breast cancer (BC) has been the most prevalent cause of cancer-associated morbidity and mortality in women. An estimated 2.3 million new cases and 685,000 female BC deaths emerged globally in 2020, which makes up a quarter of cancer diagnoses and one in six female cancer deaths [1]. Due to its complex biology and inconsistent clinical findings, breast cancer seems to be a major public health problem, even with advancements in early diagnosis and treatment modalities. Breast cancer is an extremely diverse condition that comprises several molecular subtypes with different genetic makeup and treatment outcomes. Hormone receptor-positive, human epidermal growth factor receptor 2 (HER2)-positive, and triple-negative breast cancer (TNBC) are broad categories determined by the expression of hormone receptors and human epidermal growth factor receptor 2 (HER2). While HER2-positive tumors are more invasive, they can still be treated with targeted therapies. Hormone receptor-positive breast cancer is the most widespread subtype and often responds effectively to endocrine therapy. TNBC lacks these receptors and has been linked with a poor prognosis, higher recurrence rates, and restricted treatment alternatives. Breast cancer treatment is further complicated by metastatic progression, recurrence, and drug resistance. Therapeutic efficacy is restricted, and survival rates are drastically decreased when metastasis spreads to organs such as the brain, liver, bone, and lungs. Long-term disease control remains severely compromised by the emergence of therapeutic resistance and tumor recurrence after therapy. These difficulties highlight the urgent need for more efficient and focused treatment approaches [2-4].

Current treatment landscape

Breast cancer management involves a multidisciplinary approach that includes surgery, radiotherapy, chemotherapy, endocrine therapy, targeted therapy, and, in selected patients, immunotherapy. Treatment selection is guided by tumor stage, molecular subtype, and biomarker status, with chemotherapeutic agents such as doxorubicin and paclitaxel remaining standard treatments, while HER2-targeted agents (e.g., trastuzumab) and endocrine therapies have substantially improved outcomes in HER2-positive and hormone receptor-positive breast cancers, respectively [5-11]. Despite these therapeutic advances, treatment efficacy remains limited by systemic toxicity, poor tumor selectivity, multidrug resistance, disease recurrence, and metastatic progression. These limitations underscore the need for advanced drug delivery strategies capable of improving therapeutic precision, enhancing tumor-specific drug accumulation, and minimizing off-target adverse effects.

Limitations of existing therapies and need for advanced drug delivery systems

Despite significant advancements in breast cancer management, conventional therapeutic approaches continue to face several critical limitations that affect overall treatment efficacy. Off-target cytotoxicity is one of the primary concerns because conventional chemotherapy drugs have unfavorable systemic effects on both malignant and healthy rapidly dividing cells. Another key limitation is poor bioavailability, which reduces the effective concentration of drugs at the tumor site due to rapid metabolism, low solubility, and inadequate tissue penetration. This often leads to suboptimal therapeutic responses despite adequate dosing [12].

Furthermore, multidrug resistance (MDR), in which tumor cells develop resistance through mechanisms such as drug efflux, accelerated DNA repair, and evasion of cell apoptosis, remains a significant challenge in breast cancer therapy. These resistance mechanisms significantly reduce the effectiveness of standard chemotherapeutic regimens [13]. In addition, the absence of tumor selectivity in traditional therapies leads to indiscriminate drug distribution, resulting in harm to healthy tissues and constraining therapy accuracy. Inadequate drug accumulation within tumor tissues is another consequence of this non-specific targeting, which minimizes the effectiveness of treatment [14]. These problems collectively underscore the necessity for more sophisticated and focused therapeutic approaches in breast cancer treatment.

Traditional drug delivery systems lack specificity, frequently impacting both malignant and healthy tissues. By allowing controlled and site-specific drug release, the drug delivery system aims to overcome these challenges. These mechanisms include the enhanced penetration and retention (EPR) effect. Additionally, by co-delivering multiple drugs, these systems provide combination therapy, which improves clinical outcomes and lowers drug resistance [15]. Through nanoscale delivery systems capable of interacting with biological environments at the molecular level with biological elements, nanotechnology has made significant advances in cancer therapy [16]. Nanoparticles, including liposomes, polymeric carriers, and inorganic systems, improve drug bioavailability and targeting efficiency [17]. In addition to therapeutic delivery, nanotechnology enables theranostic applications that integrate diagnosis and treatment within a single platform [18]. These approaches have expanded cancer management strategies, including gene therapy, immunotherapy, and phototherapy [19].

Rationale for hybrid nanoparticle systems

Polymer-metal hybrid nanoparticles are multifunctional nanostructures composed of an organic polymeric component integrated with an inorganic metallic component, typically arranged as a metallic core coated with a polymer shell or as a polymeric matrix incorporating metallic nanoparticles. The polymeric component provides biocompatibility, biodegradability, controlled drug loading, and sustained drug release, whereas the metallic component contributes unique physicochemical properties, including optical, photothermal, magnetic, catalytic, and imaging capabilities. By combining these complementary characteristics within a single platform, polymer-metal hybrid nanoparticles enable enhanced therapeutic efficacy, improved tumor targeting, and multifunctional theranostic applications for cancer treatment. The necessity to overcome complex biological and physicochemical obstacles that restrict the effectiveness of conventional nanocarriers in oncology essentially drives the development of hybrid nanoparticle systems. Therapeutic outcomes are severely hindered by tumor heterogeneity, complex microenvironmental factors like hypoxia and acidic pH, and biological barriers like opsonization and quick clearance [20-21].

Hybrid nanoparticles are rationally engineered to integrate complementary material properties, enabling synergistic enhancement of stability, targeting efficiency, and therapeutic performance. These systems offer enhanced structural integrity and functional adaptability by integrating organic and inorganic components, enabling precise control over drug loading and release kinetics. Additionally, multiple-stage targeting techniques including prolonged systemic circulation, enhanced tumor accumulation, and effective intracellular transport are supported by hybrid designs. Additionally, multiple therapeutic drugs with diverse modes of action can be delivered simultaneously using hybrid nanoparticles, allowing for the regulation of multiple cellular pathways and overcoming multidrug resistance. Their ability to incorporate stimuli-responsive elements further enhances spatiotemporal control of drug release, responding to internal tumor signals or external triggers. The design advantages of hybrid nanoparticle systems establish them as multifunctional platforms that integrate therapeutic, diagnostic, and prognostic capabilities, consistent with the developing paradigm of precision oncology and customized cancer treatment [21-23]. Despite substantial advances in nanoparticle-based therapeutics, the clinical translation of polymer-metal hybrid nanoparticle systems for breast cancer remains limited by challenges related to manufacturing, long-term safety, tumor heterogeneity, and regulatory approval. Although numerous experimental and translational studies have been published, a comprehensive synthesis integrating biological mechanisms, therapeutic applications, patient-centered outcomes, clinical evidence, and translational barriers is lacking.

The primary objective of this review is to provide a comprehensive and critical overview of polymer-metal hybrid nanoparticle (PMHNP) systems as emerging nanoplatforms for targeted breast cancer therapy. Specifically, this review examines the design principles, structural architectures, synthesis strategies, and surface functionalization approaches that govern the physicochemical and biological performance of these hybrid nanocarriers. It further discusses passive and active tumor-targeting mechanisms, stimuli-responsive drug delivery, and multifunctional applications, including molecular imaging, photothermal therapy, and theranostic interventions. In addition, the review summarizes recent preclinical and clinical advances; highlights the role of PMHNPs in overcoming biological barriers, multidrug resistance, and tumor heterogeneity; and critically evaluates the remaining challenges associated with biocompatibility, large-scale manufacturing, regulatory approval, and clinical translation. By integrating current evidence and identifying future research directions, this review aims to provide a comprehensive resource for researchers and clinicians developing next-generation polymer-metal hybrid nanomedicines for precision breast cancer management. This review synthesizes current preclinical, translational, and clinical evidence to provide a comprehensive overview of polymer-metal hybrid nanoparticle systems in breast cancer.

## Review

Literature search strategy and methodology

A structured literature search was conducted to identify relevant studies on polymer-metal hybrid nanoparticles for targeted breast cancer therapy. Electronic databases, including PubMed/MEDLINE, Scopus, Web of Science, ScienceDirect, and Google Scholar, were searched for articles published between January 2014 and March 2026. Seminal studies published before 2014 were also included where necessary to provide historical context and foundational information.

The search strategy employed combinations of Medical Subject Headings (MeSH) terms and free-text keywords using Boolean operators (AND/OR), including: "breast cancer," "polymer-metal hybrid nanoparticles," "metal nanoparticles," "polymeric nanoparticles," "nanomedicine," "targeted drug delivery," "theranostics," "photothermal therapy," "magnetic nanoparticles," "stimuli-responsive drug delivery," "tumor targeting," "clinical translation," and "breast cancer nano therapy."

The identified literature was critically evaluated and qualitatively synthesized to summarize current evidence, technological advances, therapeutic applications, clinical translation, challenges, and future directions. As this article was designed as a narrative review, formal systematic review procedures and quantitative evidence synthesis methods were not employed. Eligibility criteria included peer-reviewed English-language publications focusing on polymeric nanoparticles, metallic nanoparticles, and hybrid nanoparticle systems for breast cancer diagnosis and therapy. Original research articles, preclinical and clinical studies, translational investigations, systematic reviews, meta-analyses, and significant review articles were considered. Conference abstracts, editorials, duplicate records, non-peer-reviewed publications, and studies unrelated to breast cancer or polymer-metal hybrid nanoparticle applications were excluded.

Although this review primarily emphasizes literature published between 2014 and 2026, a limited number of earlier publications were intentionally included because they constitute landmark studies that established the fundamental concepts of cancer nanomedicine, including the enhanced permeability and retention (EPR) effect, nanoparticle-based drug delivery, polymeric nanocarrier design, and theranostic applications. These seminal references provide the scientific foundation necessary to contextualize recent advances in polymer-metal hybrid nanoparticle systems and their clinical translation.

The selected literature was critically evaluated and qualitatively synthesized to summarize nanoparticle design strategies, mechanisms of action, therapeutic applications, multifunctional properties, clinical evidence, translational challenges, and future research directions. As the primary objective was to provide a comprehensive and critical overview of the existing evidence, this study was designed as a narrative review supported by a structured literature search rather than a formal systematic review or meta-analysis.

Polymer-Based Drug Delivery Systems

Over the past 10 years, innovative delivery systems based on synthetic and natural polymers with improved pharmacokinetic and therapeutic potential for cancer treatment have expanded significantly. Polymer-based drug delivery systems have garnered significant interest in breast cancer treatment due to their biodegradability, biocompatibility, and capacity for regulated and sustained drug release. These techniques improve the pharmacokinetic profile of conventional chemotherapeutic drugs and reduce systemic toxicity by stimulating drug accumulation at the tumor site [24]. Poly(lactic-co-glycolic acid) (PLGA) is one of the most extensively investigated biodegradable polymers because of its excellent biocompatibility, biodegradability, and tunable drug-release properties. PLGA has been widely used in FDA-approved pharmaceutical formulations and medical devices, making it an attractive material for nanoparticle-based drug delivery systems.

PLGA nanoparticles have been successful at encapsulating anticancer medications, specifically doxorubicin and paclitaxel, leading to longer circulation times and improved therapeutic outcomes compared to other delivery methods. Poly(lactic-co-glycolic acid) (PLGA) is a very effective biodegradable polymer, as its breakdown results in the metabolite monomers, lactic acid and glycolic acid. The endogenous nature and facile metabolism of these two monomers via the Krebs cycle result in low systemic toxicity when using PLGA for drug delivery or biomaterial applications [25].

Polyethylene glycol (PEG) is a common choice for modifying nanoparticle surfaces because it creates a stealth effect that reduces the likelihood that an opsonized surface will be rapidly removed from circulation by cells of the mononuclear phagocyte system. This PEGylation process prolongs systemic circulation time and enhances passive tumor targeting through the enhanced permeability and retention (EPR) effect [26]. In breast cancer treatment, PEGylated nanocarriers exhibit enhanced bioavailability, reduced off-target toxicity, and superior tumor accumulation relative to non-PEGylated systems. Recent research on PEGylated nanoliposomes containing anticancer drugs like doxorubicin and paclitaxel has demonstrated improved therapeutic efficacy and decreased cardiotoxicity, especially in HER2-positive and triple-negative breast cancer models. Additionally, under physiological conditions, PEG-coated nanoparticles enhance colloidal stability and reduce aggregation, allowing for more efficient and reliable drug administration. However, excessive PEG concentrations can reduce intracellular drug release and cellular absorption a phenomenon known as the "PEG dilemma," which remains a critical aspect in nanoparticle formulation [27].

Chitosan, a biodegradable and biocompatible polysaccharide derived from chitin, has been thoroughly investigated as a potential nano vehicle for cancer treatment. Chitosan exhibits potential in cancer therapy, particularly as a solubilizing agent, drug stabilizer, and for controlled drug release, which has served as a versatile platform for targeting, stimulus-responsive release, or application in image-guided medicine [28]. It offers multiple advantages, including mucoadhesion, enhanced drug-loading efficiency, and optimized cellular internalization owing to its positive surface charge. Researchers have investigated chitosan-based nanoparticles for the simultaneous delivery of chemotherapeutic agents and nucleic acids. This approach is especially useful for addressing multidrug resistance in breast cancer cells [29].

Notwithstanding these benefits, polymeric systems exhibit significant limitations, such as rapid drug release, inadequate stability in physiological environments, and restricted active targeting capacity. Their lack of imaging capabilities or reactivity to external stimuli limits their therapeutic accuracy. These obstacles have led to the development of hybrid nanoparticle systems that combine metallic elements with polymeric carriers to enhance targeting and multifunctional efficacy.

Metal Nanoparticles in Cancer Therapy

Metallic nanoparticles (MNPs) have emerged as a promising tool for BC diagnosis and therapy with the development of nanotechnology. MNPs have demonstrated significant potential to overcome the drawbacks of traditional therapeutic and diagnostic techniques due to their distinctive physical and chemical properties, such as high surface area, controlled size, surface modification, and unique optical, magnetic, or catalytic properties. Their tailored physicochemical properties enable applications in targeted drug delivery, imaging, photothermal therapy, and theranostics, while their nanoscale size enables superior tumor accumulation [30]. The most studied metallic nanomaterials for the treatment of breast cancer are gold nanoparticles (AuNPs), silver nanoparticles (AgNPs), and iron oxide nanoparticles (IONPs) [31].

Gold nanoparticles are particularly appealing due to their superior biocompatibility, surface plasmon resonance, and ease of surface alterations with drug molecules, antibodies, and targeting ligands [32]. Their high photothermal conversion efficiency allows targeted photothermal ablation with near-infrared light exposure, minimizing toxicity to nearby healthy tissues. In HER2-positive breast cancer, trastuzumab-functionalized AuNPs have demonstrated higher receptor-specific binding and improved internalized drug absorption [33]. AuNPs exhibit excellent physicochemical stability and have generally demonstrated favorable biocompatibility in many preclinical studies. However, their biological behavior and safety profile depend on factors such as particle size, shape, surface chemistry, dose, and biodistribution and therefore cannot be assumed solely from the biocompatibility of bulk gold. Bulk gold is considered a biocompatible material for several medical applications. However, the safety of gold nanoparticles cannot be inferred solely from bulk gold, as their biological interactions are strongly influenced by particle size, morphology, surface functionalization, and physicochemical properties [34].

Several recent studies have shown that targeted delivery of gold nanoparticles in combination with mild hyperthermia produces a synergistic effect that enhances the effectiveness of chemotherapy by improving the localized destruction of cancers while minimizing damage to adjacent healthy tissue. Development of breast cancer-targeting functionalized nanoparticle systems has led to improved cellular uptake, greater drug matrix accumulation at the tumor site, and greater sensitivity to both thermal and chemotherapy treatments. The delivery of anticancer agents in combination with photothermal properties has also resulted in additive therapeutic effects through a much greater inhibitory effect on tumor growth than that seen with traditional chemotherapy alone [35]. The development of image-guided and multimodal therapeutic systems has been aided by developments in nanoparticle surface modification and targeting techniques, which have further enhanced selective interaction with breast cancer cells. The widespread clinical application of metallic nanoparticle systems in the treatment of breast cancer is yet constrained because of concerns regarding long-term biodistribution, biodegradation, systemic clearance, and the risk of toxicity, despite promising therapeutic results. Silver nanoparticles (AgNPs) have emerged as promising metallic nanomaterials in breast cancer therapy because of their effective cytotoxic and apoptosis-inducing abilities. Silver nanoparticles exhibit significant anticancer activity mainly through the generation of reactive oxygen species (ROS), which induce oxidative stress, mitochondrial dysfunction, DNA damage, and apoptosis in breast cancer cells. However, their therapeutic application requires careful dose optimization because excessive ROS generation may also affect normal tissue [36]. Additionally, AgNPs indicate the ability to suppress tumor growth and angiogenesis, especially in aggressive subtypes such as triple-negative breast cancer [37].

Silver nanoparticles reported a specific anticancer activity in breast cancer models, including Michigan Cancer Foundation-7 (MCF-7) and MD Anderson-MB-231 (MDA-MB-231) cells, by disrupting cellular redox reactivity and triggering reactive oxygen species-mediated cell death [38]. Additionally, biosynthesized and functionalized AgNPs have demonstrated superior cellular uptake and targeted delivery, revealing their potential application in combination therapies and treatment approaches based on nanomedicine [39]. AgNPs interfere with metabolic regulation, cell cycle progression, and mitochondrial homeostasis, which inhibits tumor cell survival and proliferation, based on proteomic and metabolomic research [40]. Despite these promising findings, concerns regarding nanoparticle-associated toxicity, nonspecific tissue accumulation, and long-term biosafety continue to limit their progression toward large-scale clinical application in breast cancer treatment.

Iron oxide nanoparticles possess superparamagnetic properties that make them highly valuable for magnetic targeting, magnetic resonance imaging (MRI), and hyperthermia-based therapy. These nanoparticles can be guided externally using magnetic fields to improve site-specific drug accumulation and reduce systemic toxicity. Research has shown that iron oxide nanoparticle-induced magnetic hyperthermia treatments can specifically kill tumor cells when heated locally with high-frequency alternating and rotating magnetic fields (radiofrequency/alternating magnetic field (RF/AMF)). This provides a highly precise method of effectively killing tumor cells, while causing minimal damage to surrounding healthy tissue. These nanoparticles have also been investigated as drug delivery systems that may enhance intratumoral accumulation of chemotherapeutic drugs and improve treatment response in breast cancer models. Additionally, their magnetic properties enable simultaneous application in conventional magnetic resonance imaging (MRI), facilitating real-time therapeutic efficacy monitoring and image-guided therapy [41]. Research on translational and preclinical levels continues to indicate that the use of iron oxide nanoparticle-theranostic combinations to enhance chemotherapeutics, hyperthermic and targeted delivery methods, and combinations thereof, would aid in addressing breast cancer. In addition, concerns about safety, toxicity of the nanoparticles, degradation of the nanoparticles over time, safety testing for the nanoparticles, and also biodistribution of the nanoparticles present challenges that will hinder their use in clinical practice across a broader population.

However, fully integrated polymer-metal hybrid nanoparticle systems have not yet achieved significant clinical trial representation. This translational gap is primarily due to challenges including complex synthesis, batch-to-batch variability, long-term toxicity concerns, and difficulties in large-scale manufacturing. Additionally, stringent regulatory requirements imposed by agencies such as the U.S. Food and Drug Administration and the European Medicines Agency further complicate clinical approval pathways. Ongoing research efforts are focused on improving the clinical feasibility of hybrid nanoplatforms through enhanced biocompatibility, scalable synthesis methods, and standardized safety evaluation protocols. These advancements are expected to facilitate the successful translation of hybrid nanoparticle systems from preclinical studies to clinical applications.

Clinically approved nanomedicines and their translational significance

The translational potential of nanomedicine in oncology has been proven by the successful clinical approval of several anticancer formulations based on nanoparticles. One of the most significant advances in translational nanomedicine involves the clinical approval of anticancer treatments based on nanoparticles. Despite significant preclinical research, relatively few nanoparticle-derived technologies have been effectively translated into clinical applications, providing a foundation for the future development of hybrid nanoplatforms. Among the most established examples are nanoparticle albumin-bound paclitaxel (Abraxane®, Bristol-Myers Squibb, Princeton, New Jersey, USA) and pegylated liposomal doxorubicin (Doxil®, Janssen Products, LP, Horsham, Pennsylvania, USA/Caelyx®, Baxter Holding B.V., Utrecht, Netherlands), both of which achieved clinical success by addressing significant issues with traditional chemotherapy. Their commercial success showed that nanoparticle systems can boost pharmacokinetic efficacy, diminish systemic toxicity, and improve therapeutic index while maintaining effective anticancer activity.

Pegylated liposomal doxorubicin (Doxil®) was the first FDA-approved nanomedicine for the treatment of solid tumors and remains one of the most therapeutically successful liposomal formulations in oncology [42]. In comparison with conventional doxorubicin therapy, recent clinical and translational investigations have shown that this nanoformulation considerably reduces anthracycline-associated cardiotoxicity and enhances therapeutic tolerance [43]. Doxorubicin's liposomal encapsulation reduces exposure to healthy tissues to unbound doxorubicin while enhancing drug distribution and circulatory stability. Moreover, the formulation was associated with a lower incidence of severe cardiovascular adverse reactions, which continue to be a major disadvantage of traditional anthracycline treatment. These findings indicate that liposomal nanoparticle systems possess the ability to improve therapeutic tolerance in clinical oncology settings while preserving robust anticancer activity [44].

In a similar manner, nanoparticles bound to albumin have achieved significant clinical relevance through overcoming multiple limitations of existing solvent-based paclitaxel therapy. The albumin nanoparticle formulation has enhanced drug solubility and allows for improved transcytosis through the endothelium and accumulation of drug at the tumor site, without using toxic solvent carriers like Cremophor (BASF SE, Ludwigshafen, Germany). Recent research on triple-negative and metastatic breast cancer showed that nab-paclitaxel increases tumor penetration, improves therapeutic response, and lessens hypersensitivity signs which are frequently linked to traditional paclitaxel formulations. In neoadjuvant breast cancer cases, nab-paclitaxel-containing regimens exhibited higher pathological complete response rates and superior compatibility with combination therapy, including immunotherapeutic drugs [45,46]. The relatively straightforward nanoparticle synthesis, uniform pharmacokinetic characterization, and scalable production method substantially facilitated its effective clinical translation and regulatory clearance in oncology.

Myocet® (CHEPLAPHARM Arzneimittel GmbH, Greifswald, Germany), a non-pegylated liposomal derivative of doxorubicin, was developed to reduce the cardiotoxic effects often linked to standard anthracycline therapy. Clinical research on metastatic breast cancer revealed superior cardiac safety while maintaining similar anticancer activity, thus highlighting the relevance of liposomal encapsulation for promoting therapeutic tolerance [47]. In contrast to pegylated liposomes, Myocet® demonstrates a lower circulation time, while ensuring an optimum equilibrium between efficacy and toxicity for particular patient groups.

Genexol-PM® (Samyang Biopharmaceuticals, Seoul, South Korea), a polymeric micelle formulation of paclitaxel, is another therapeutically significant nanomedicine authorized in various Asian nations for the treatment of metastatic breast cancer [48]. Also, polymeric micellar administration promotes drug stability and circulation dynamics, leading to better tolerance and therapeutic efficacy.

Onivyde® (Ipsen Biopharmaceuticals, Inc., Cambridge, Massachusetts, USA) and other liposomal irinotecan formulations have shown translational effectiveness in solid tumor therapy. Irinotecan encapsulation in liposomal vesicles improves pharmacokinetic performance and intratumoral drug retention by extending systemic circulation and permitting prolonged drug release [49]. Current research continues to evaluate its therapeutic effect in breast cancer and other advanced malignancies, although it is primarily approved for pancreatic cancer.

Nanomedicine for oncology has been developed via the addition of polymeric nanoparticle systems, such as CRLX101 (Cerulean Pharma Inc, Cambridge, Massachusetts, USA). CRLX101 is a cyclodextrin polymeric nanoparticle carrying camptothecin, which was created to increase drug solubility, extend the duration of circulation time, and target tumor accumulation [50]. Previous clinical studies of breast cancer and refractory solid tumors suggested positive pharmacokinetic and therapeutic properties; however, there are still numerous issues associated with inconsistencies in patient response and issues related to large-scale manufacturing which continue to hinder clinical use.

Liposomal and polymeric nanoparticle platforms have been thoroughly studied in metastatic and HER2-positive breast cancer for their ability to overcome multidrug resistance and further improve intratumoral drug delivery [51]. In addition, multifunctional nanocarriers that combine imaging and therapeutic purposes have emerged as a promising technique for targeted breast cancer therapy and theranostic applications [52]. Despite these advances, the clinical translation of many nanoparticle systems remains limited by challenges associated with large-scale manufacturing, formulation consistency, biological heterogeneity, and long-term safety evaluation [48]. These opportunities and limitations of representative nanomedicine platforms are summarized in Table 1.

**Table 1:** Overview of Nanomedicine Platforms for Cancer Therapy: Nanoparticle Types, Therapeutic Components, Clinical Applications, Advantages, Challenges, and Clinical Status PEG: polyethylene glycol; HER2: human epidermal growth factor receptor 2; NSCLC: non-small cell lung cancer.

Nanomedicine Platform	Nanoparticle Type	Encapsulated Drug/Functional Component	Cancer Type Clinical Application	Clinical Status	Major Clinical Advantages	Key Challenges	Reference
Doxil®	PEGylated liposomal nanoparticle	Doxorubicin	Metastatic breast cancer, ovarian cancer	FDA-approved	Reduced cardiotoxicity, prolonged circulation, lower systemic toxicity	Hand-foot syndrome, limited active targeting capability	[42]
Abraxane® (nab-paclitaxel)	Albumin-bound nanoparticle	Paclitaxel	Metastatic breast cancer, NSCLC, pancreatic cancer	FDA-approved	Solvent-free formulation, improved tumor penetration, reduced hypersensitivity reactions	Peripheral neuropathy, high treatment cost	[45]
Liposomal Irinotecan (Onivyde®)	Liposomal nanoparticle	Irinotecan	Solid tumors	FDA-approved	Enhanced pharmacokinetics, sustained release	Gastrointestinal toxicity, neutropenia	[49]
Myocet	Non-PEG liposome	Doxorubicin citrate	HER2-positive metastatic breast cancer	Approved in Europe and Canada	Lower cardiotoxicity compared with free doxorubicin, improved tolerability	Limited availability, lower tumor targeting efficiency than PEGylated system	[47]
Genexol-PM®	Polymeric micelle	Paclitaxel	Advanced and metastatic breast cancer	Approved in South Korea	Cremophor-free formulation, enhanced solubility, improved safety profile	Stability issues, limited global regulatory approval	[48]
CRLX101	Cyclodextrin-based polymeric nanoparticle	Camptothecin	Breast cancer and refractory solid tumors	Phase II clinical trial	Improved drug solubility, prolonged circulation, enhanced tumor accumulation	Hematologic toxicity and variable patient response	[50]
AuroShell® (Nanospectra Biosciences, Inc., Houston, Texas, USA) Nanoparticles	Silica-gold nanoshells	Photothermal gold nanoparticle system	Breast cancer and solid tumor photothermal ablation	Early clinical evaluation	Localized photothermal tumor destruction, image-guided therapy potential	Long-term biodistribution and clearance concerns	[53]
Iron oxide nanoparticles (Ferumoxytol-based systems)	Magnetic iron oxide nanoparticle	Magnetic hyperthermia/imaging agents	Breast cancer theranostics and MRI-guided therapy	Phase I/II investigations	MRI compatibility, magnetic targeting, hyperthermia-mediated apoptosis	Concerns regarding accumulation and biosafety	[54]
Magnetic hyperthermia Nanoplatforms	Functionalized iron oxide nanoparticles	Heat-generating magnetic system	Breast cancer targeted hyperthermia	Translational/investigational	Localized apoptosis induction, enhanced chemotherapy sensitivity	Optimization of magnetic field exposure required	[55]
HER2-targeted polymeric nanocarriers	Polymeric nanoparticle	Anti-HER2 therapeutics	HER2-positive breast cancer	Experimental/translational	Improved receptor-specific targeting, enhanced cellular uptake	Limited clinical validation	[56]
Multifunctional theranostic nanoparticles	Hybrid lipid-polymer systems	Imaging + chemotherapeutic agents	Breast cancer precision medicine	Preclinical/early translational	Combined diagnosis and therapy, personalized treatment potential	Complex manufacturing and regulatory approval	[57]

Clinical Relevance

A crucial obstacle in nanoparticle-based cancer treatment is the persistent gap between mechanistic comprehension, nanoparticle design methods, and preclinical effectiveness, in relation to their transformation into clinically significant results. Although nanomedicine-based research has grown rapidly over the past decade, most recent developments have been limited to experimental conditions, with only a few reaching clinical applications. The increased permeability and retention (EPR) effect, accompanied by active targeting techniques like ligand-receptor interactions and stimuli-responsive drug release, represents the core mechanistic foundations of nanoparticle delivery systems. The clinical prediction of these systems remains restricted, considering their constant confirmation in both in vitro and in small animal experiments. There is increasing evidence showing that the EPR effect varies greatly among patients and tumor types due to differences in interstitial fluid pressure, vascular permeability, and tumor microenvironment features [58,59]. The accumulation of nanoparticles in cancer cells in humans is often significantly lower and more variable compared to animal models, which reduces the effectiveness of treatment. In addition, the new research challenges the traditional EPR-based paradigm, as it shows that active transendothelial transport mechanisms are required for the delivery of nanoparticles, rather than just passive leakage [60]. 

Advances in nanoparticle design, including lipid-based carriers (such as liposomes), polymeric systems, dendrimers, micelles, and inorganic nanostructures, have significantly improved drug delivery characteristics, including enhanced solubility, physicochemical stability, and prolonged systemic circulation. These mechanisms have been developed to improve the therapeutic index by facilitating controlled or stimulus-responsive drug release and optimizing pharmacokinetics. Notwithstanding these engineering breakthroughs, successful clinical translation remains constrained. An important contributing factor is the interaction of nanoparticles with biological systems, particularly the formation of protein coronas, which dynamically modify the surface properties of nanoparticles and can significantly alter biodistribution, targeting efficiency, and cellular uptake [61]. Clinical standardization is further hampered by other obstacles such as large-scale manufacturing challenges, batch-to-batch variability, regulatory complexity, and concern about long-term toxicity and clearance.

Clinical Translation Perspective

The clinical success of nanoparticle-based drug delivery systems in breast cancer therapy is exemplified by approved formulations such as PEGylated liposomal doxorubicin (Doxil®/Caelyx®) and albumin-bound paclitaxel (Abraxane®), which exhibit improved pharmacokinetic profiles, enhanced tumor accumulation through optimized biodistribution, and reduced systemic toxicity compared with their conventional formulations. These clinically approved nanomedicines provide compelling evidence for the translational potential of nanotechnology in oncology by demonstrating that rationally engineered nanoscale delivery systems can improve the therapeutic index, enhance treatment efficacy, and mitigate dose-limiting adverse effects. Their successful clinical implementation has also laid an important foundation for the development and translation of next-generation multifunctional polymer-metal hybrid nanoparticle platforms for targeted breast cancer therapy [18,37,38].

Despite these advances, polymer-metal nanoparticle hybrid systems, while highly promising in preclinical studies, have not yet achieved significant clinical translation. This gap underscores critical challenges, including complex and non-scalable synthesis methods, batch-to-batch variability, insufficient long-term safety data, and difficulties in reproducibility under clinical-grade manufacturing conditions. Furthermore, the intricate design of hybrid systems often complicates their pharmacokinetic and toxicological evaluation, delaying their progression into human trials.

From a regulatory standpoint, approval pathways for such advanced nanotherapeutics remain stringent and not fully standardized. Regulatory bodies such as the U.S. Food and Drug Administration and the European Medicines Agency require comprehensive characterization, including physicochemical properties, biodistribution, biodegradability, and long-term toxicity profiles. The absence of universally accepted regulatory frameworks specifically tailored for hybrid nanomaterials further complicates their clinical approval. Addressing these translational and regulatory challenges is essential to bridge the gap between promising laboratory findings and successful clinical implementation. The clinical data presented in Table 2 demonstrate that nanoparticle-based drug delivery systems have shown significant potential to improve the safety profile, pharmacokinetic characteristics, and therapeutic efficacy of breast cancer treatments, thereby supporting their clinical translation into oncology practice.

**Table 2:** Selected Clinical Trials of Nanoparticle-Based Systems in Breast Cancer and Related Solid Tumors NCT: National Clinical Trial.

NCT Number	Intervention	Phase	Indication	Key Outcomes	References
NCT00848042	Gold nanoshell-mediated photothermal therapy (AuroLase®, Nanospectra Biosciences, Inc., Houston, Texas, USA)	Phase I	Solid tumors (including breast cancer)	Demonstrated a favorable safety profile, the feasibility of photothermal ablation, and localized tumor control in early-stage evaluation	[53]
NCT01644890	Iron oxide nanoparticles (magnetic hyperthermia)	Phase I/II	Recurrent breast cancer/solid tumors	Showed acceptable safety, controlled tumor heating, and preliminary therapeutic efficacy in combination with other treatments	[41]
NCT00848042	Superparamagnetic iron oxide nanoparticles (SPIONs) for MRI	Phase I	Breast cancer imaging	Improved tumor detection sensitivity and good biocompatibility, supporting diagnostic utility	[30,41]
NCT00442260	Abraxane +Doxil	Phase I/II	Metastatic breast cancer	Demonstrated enhanced response rates, improved drug delivery, and reduced hypersensitivity compared to conventional paclitaxel	[45]

In contrast, metallic nanoparticle platforms, including gold nanoshells and iron oxide nanoparticles, remain largely in early-phase (Phase I/II) clinical trials, where they have demonstrated promising safety profiles and preliminary efficacy but require further validation in larger cohorts [39-41]. Importantly, despite substantial advances in preclinical research, no polymer-metal hybrid nanoparticle formulation has yet received approval from the U.S. Food and Drug Administration (FDA) or the European Medicines Agency (EMA) for the treatment of breast cancer. This highlights a significant translational gap between experimental innovation and clinical implementation, emphasizing the need for standardized manufacturing processes, comprehensive safety evaluations, and robust clinical validation. Notably, polymer-metal hybrid nanoparticle systems are absent from advanced clinical trials, highlighting a critical translational gap between preclinical innovation and clinical application. This gap is primarily attributed to challenges in large-scale synthesis, reproducibility, long-term toxicity assessment, and regulatory approval pathways [18].

Patient-oriented therapeutic outcomes

In the evaluation of targeted breast cancer therapies involving polymeric hybrids with metal nanoparticles in their composition, patient-centered outcomes have become an increasingly important factor. Modern oncologic therapy regimens focus not only on traditional measures such as treatment efficacy but also on the quality of life (QoL), limiting or eliminating systemic toxicity, adherence to treatment regimens, improving psychological well-being, and supporting long-term survival. The selective tumor targeting, controlled drug release, and reduced off-target toxicity rendered feasible by nanotechnology-based drug delivery systems make them an appealing substitute to traditional chemotherapy in these circumstances [62-64].

One of the most prominent patient-centered benefits of nanoparticle-mediated therapy is the reduction in chemotherapy-associated side effects. Traditional cytotoxic drugs such as doxorubicin, paclitaxel, and vinorelbine often produce severe systemic toxicities including cardiotoxicity, neurotoxicity, alopecia, nausea, and myelosuppression, which substantially impair patient quality of life and frequently lead to treatment discontinuation. Polymeric and metallic nanoparticle hybrid systems improve pharmacokinetic profiles by enhancing tumor-specific accumulation through the enhanced permeability and retention (EPR) effect and receptor-mediated targeting. Consequently, lower systemic drug exposure can reduce toxicity while maintaining therapeutic efficacy [65].

Nanoparticle-based formulations have shown significant improvements in patient acceptance and safety in clinical translation studies. Nanoparticle albumin-bound paclitaxel (nab-paclitaxel), a therapeutically effective nanomedicine formulation for breast cancer treatment, has demonstrated decreased hypersensitivity and the removal of toxic solvent requirements compared to traditional paclitaxel formulations. Higher tolerability allows for the administration of more powerful dosages with less additional support (e.g., antiemetics) and enhances the convenience of treatment for patients along with increased patient satisfaction [66]. Patient-centered outcomes comprise advances in progression-free survival, symptom management, and maintenance of functional status. Targeted nanocarrier systems developed for HER2-positive and triple-negative breast cancer subtypes have shown increased intracellular absorption and greater anticancer efficacy, potentially leading to improved disease management and extended survival rates. Moreover, multifunctional hybrid nanoparticles that combine therapeutic and diagnostic functions may provide real-time assessment of therapeutic efficacy, allowing for individualized treatment modifications and reducing unnecessary toxicity [67].

Reduction in negative outcomes, shorter length of time needing to be in the hospital, and increased efficacy of therapy all help improve patient self-confidence and mental health while receiving long-term cancer therapy [68]. Due to their sustained and regulated drug release properties, nanomedicine-based targeted therapies may help lessen the burden of repeated dosage regimens, improving patient convenience and overall treatment adherence.

Despite these advantages, numerous hurdles persist until comprehensive clinical adoption can completely enhance patient-centered benefits. Variability in nanoparticle biodistribution, manufacturing complexity, high treatment costs, and poor long-term safety evidence continue to restrict accessibility and clinical application. Furthermore, numerous current studies tend to focus on drug efficacy rather than standardized patient-reported outcome measures (PROMs), emphasizing the necessity for future clinical trials to incorporate complete quality-of-life assessments and survival rate analyses. Polymer-metal nanoparticle hybrid systems have been developed as an innovative means to deliver targeted therapies for breast cancer, allowing for increased treatment precision while simultaneously addressing patient-centered advances in care. The continued development of these technologically advanced forms of therapy will require translational and clinical research that incorporates both biological efficacy and patient-reported outcomes.

Tumor Microenvironment

Breast cancer development is influenced by numerous factors within the tumor microenvironment (TME). The TME of primary breast cancer consists of diverse cellular and non-cellular components, including cancer-associated fibroblasts (CAFs), immune cells, extracellular matrix (ECM) proteins, abnormal blood vessels, hypoxic regions, and an acidic microenvironment. These components create significant barriers to nanoparticle penetration and therapeutic efficacy. These components create significant barriers to the penetration and effectiveness of nanoparticles as therapeutic agents. Therefore, it is important to understand these different TME components and how they will impact the clinical advancement of polymer-metal hybrid nanoparticle systems [69,70]. Hypoxia is one of the major attributes of TMEs and is highly correlated with resistance to therapy. Fast-growing tumors develop along with their abnormal vasculature (blood vessels) leading to reduced amounts of oxygen reaching the inside of solid tumors. This activates hypoxia-inducible factors (HIFs) that cause gene expression changes that lead to angiogenesis, metastasis, immune evasion, and resistance to both chemotherapy and radiotherapy [71].

Hypoxic conditions also limit drug diffusion and reduce therapeutic responsiveness in breast cancer patients. To overcome this challenge, hypoxia-responsive nanoparticles have been developed to selectively release drugs within oxygen-deficient tumor regions, thereby improving targeting efficiency and antitumor activity. The acidic pH of the TME, which is brought about by increased glycolysis and lactate accumulation in tumor tissues, is another significant characteristic. Because pH-responsive polymeric nanoparticles can accomplish regulated and site-specific drug release within tumor cells while remaining stable under physiological conditions, this acidic environment is extremely relevant for nanomedicine applications. These systems lessen systemic toxicity and enhance intracellular drug accumulation [69,72].

Beyond hypoxia and acidic pH, several cellular and structural components of the tumor microenvironment (TME) significantly influence the therapeutic performance of polymer-metal hybrid nanoparticle systems. Cancer-associated fibroblasts (CAFs) promote tumor progression by remodeling the extracellular matrix (ECM), increasing tissue stiffness, and limiting nanoparticle penetration into tumor tissues. Similarly, tumor-associated macrophages (TAMs), particularly the immunosuppressive M2 phenotype, contribute to immune evasion, angiogenesis, and therapeutic resistance, thereby reducing treatment efficacy. Immune checkpoint interactions, including the programmed cell death protein 1/programmed death-ligand 1 (PD-1/PD-L1) axis, further suppress antitumor immune responses and create an environment conducive to tumor survival. In addition, dense ECM barriers restrict nanoparticle diffusion and hinder uniform intratumoral drug distribution. Consequently, the development of multifunctional hybrid nanoplatforms capable of modulating CAF activity, reprogramming TAMs, overcoming immune checkpoint-mediated suppression, and enhancing ECM penetration represents a promising strategy for improving therapeutic outcomes and advancing precision breast cancer nanomedicine [62-64,69-72]. Overall, targeting hypoxia, acidic pH, and stromal barriers within the TME may significantly improve the therapeutic performance and clinical translation of polymer-metal nanoparticle hybrid systems in breast cancer therapy.

Manufacturing and scale-up limitations

The clinical application and commercialization of polymer-metal nanoparticle hybrid systems in breast cancer treatment are still significantly hampered by manufacturing issues [50]. Industrial application remains constrained by challenges with batch repeatability, large-scale production, physicochemical stability, and quality control, despite promising preclinical results. Batch-to-batch reproducibility is one of the most critical concerns in nanoparticle manufacturing [73]. Minor modifications in temperature, pH, solvent, mixing speed, and precursor composition can significantly alter the size, shape, surface charge, and ability of nanoparticles to transport and release drugs. Regulatory clearances and the shift to clinical usage may become more difficult as a result of these kinds of alterations since they may increase variability in therapeutic efficacy, body distribution, and safety [74].

The successful clinical translation of polymer-metal hybrid nanoparticle systems depends not only on their therapeutic performance but also on reproducible large-scale manufacturing, robust quality control, long-term physicochemical stability, regulatory compliance, and cost-effective production. Addressing these challenges is essential for the successful commercialization and widespread clinical adoption of next-generation nanomedicines. Furthermore, maintaining uniform particle dispersion, physicochemical stability, and long-term storage stability remains technically challenging. Many nanoparticle formulations demonstrate excellent performance at the laboratory scale but encounter significant obstacles during industrial-scale production due to variations in hydrodynamic conditions, solvent evaporation rates, mixing efficiency, and process reproducibility. Traditional laboratory fabrication methods, including solvent evaporation, nanoprecipitation, and emulsion-based techniques, are often difficult to reproduce consistently under large-scale manufacturing conditions [75-78].

Safety concerns

The clinical translation of polymer-metal nanoparticle hybrid systems for the treatment of breast cancer continues to be significantly hampered by safety issues. These nanocarriers enhance therapeutic efficacy and targeted drug delivery, but their long-term biological interactions and possible toxicities are yet unclear [78]. Accumulation of metallic nanoparticles (NPs) within essential organs after multiple doses is a potential hazard. Metallic components, such as gold, silver, and iron oxide nanoparticles, may remain in the body for extended periods of time as they have slow rates of biodegradation and clearance [79]. Long-term patient safety may be impacted by persistent accumulation, which can cause oxidative stress, inflammation, organ damage, and disrupted cell signaling pathways [34].

Immunogenicity represents a significant safety issue linked to nanoparticle-based therapies. Nanoparticles might interact with immune cells and stimulate complement mechanisms, inflammatory cytokines, and immunologically mediated hypersensitivity responses. The surface characteristics and formation of a protein corona may additionally affect immune recognition and influence a nanoparticle interaction within the cellular microenvironment [80]. In some cases, repeated exposure to nanomaterials may trigger chronic immune activation or immunosuppression, potentially compromising treatment safety and efficacy. Additionally, during clinical translation, the prediction of nanoparticle-associated toxicities can be complicated by patient-specific variation in immune responses. However, before polymer-metal nanoparticle hybrid systems are widely used in clinical settings for the management of breast cancer, extensive long-term toxicity studies and standard safety evaluation procedures remain necessary.

Discussion

Breast cancer remains a biologically heterogeneous disease characterized by molecular diversity, therapeutic resistance, and variable clinical outcomes. Despite significant advances in chemotherapy, endocrine therapy, and targeted agents, the clinical management of advanced disease continues to be constrained by systemic toxicity, off-target effects, and the emergence of multidrug resistance. These limitations have driven the development of nanotechnology-based drug delivery systems aimed at improving therapeutic precision and reducing adverse effects.

The clinical relevance of nanoparticle-based therapeutics is already established through approved formulations such as Doxil and Abraxane. These agents demonstrate that nanoscale drug engineering can significantly improve pharmacokinetics, enhance tumor accumulation, and reduce systemic toxicity. For example, liposomal encapsulation of doxorubicin has been associated with reduced cardiotoxicity, while albumin-bound paclitaxel improves solubility and minimizes hypersensitivity reactions compared to conventional taxanes. These clinically validated systems provide strong evidence that nanomedicine can successfully transition from bench research to real-world oncology practice.

In contrast, polymer-metal nanoparticle hybrid systems remain largely confined to preclinical and early translational stages. While experimental studies demonstrate promising outcomes, including improved tumor targeting, enhanced intracellular drug delivery, and multimodal functions such as photothermal therapy and imaging, these findings have not yet translated into late-phase clinical trials. This represents a critical translational gap between laboratory innovation and clinical implementation. Importantly, most reported clinical investigations involving metallic nanoparticles, such as gold nanoshells and iron oxide-based systems, have been conducted in Phase I or Phase I/II settings and are typically evaluated in solid tumors, including breast cancer cohorts, rather than breast cancer-exclusive populations. These studies consistently report acceptable safety profiles along with preliminary efficacy, but robust survival benefits remain unconfirmed.

Several barriers continue to limit clinical translation of hybrid nanoplatforms. First, the complexity of their multi-component architecture introduces challenges in reproducible large-scale manufacturing and batch-to-batch consistency. Second, an incomplete understanding of long-term biodistribution, degradation, and potential accumulation, particularly of metallic components, raises safety concerns. Third, the integration of multiple functional domains within a single system complicates pharmacokinetic modelling and regulatory evaluation.

From a regulatory perspective, nanomedicine development is governed by stringent frameworks established by agencies such as the U.S. Food and Drug Administration and the European Medicines Agency, which require comprehensive characterization of physicochemical properties, immunogenicity, toxicity, and in vivo behavior. However, the absence of harmonized, nanoparticle-specific regulatory guidelines for hybrid systems remains a major bottleneck. This lack of standardization leads to variability in preclinical reporting and delays clinical progression. Representative comparative characteristics of polymeric, metallic, and polymer-metal hybrid nanoparticle systems for targeted breast cancer therapy are summarized in Table 3. These findings highlight ongoing progress toward improving efficacy, reducing systemic toxicity, and enhancing translational potential in breast cancer management.

**Table 3:** Comparative Characteristics of Polymeric, Metallic, and Polymer-Metal Hybrid Nanoparticle Systems for Targeted Breast Cancer Therapy PLGA: poly(lactic-co-glycolic acid); PEG: polyethylene glycol; NPs: nanoparticles; EPR: enhanced penetration and retention. The comparative rankings presented in this table are based on the collective findings reported across the cited literature and are intended to provide a qualitative overview of the relative advantages and limitations of each nanoparticle platform rather than absolute quantitative measurements.

Parameter	Polymeric Nanoparticles	Metallic Nanoparticles	Polymer-Metal Hybrid Nanoparticles	References
Composition	Biodegradable natural or synthetic polymers (e.g., PLGA, PEG, chitosan)	Inorganic metallic cores composed of gold, silver, iron oxide, or related materials	Integrated architectures combining polymeric matrices with metallic cores or shells	[20,24-31]
Drug encapsulation efficiency	High capacity for incorporation of hydrophilic and hydrophobic therapeutics	Generally limited loading capacity due to restricted surface area and carrier architecture	Enhanced loading through synergistic polymeric encapsulation and metallic surface functionalization	[20,24,25,57]
Drug release kinetics	Programmable and stimuli-responsive release profiles	Predominantly diffusion-dependent release with limited control	Controlled and externally triggerable release behavior	[24-25,57]
Targeted delivery performance	Surface modification enables active and passive tumor targeting	Primarily dependent on ligand conjugation and EPR-mediated accumulation	Improved targeting through combined surface engineering and multifunctional design	[20,24,51,62]
Photothermal and hyperthermic activity	Minimal intrinsic photothermal conversion	Efficient light-to-heat conversion and magnetic hyperthermia potential	Enhanced therapeutic heating combined with controlled drug delivery	[20,30-31,41,57]
Biocompatibility and biodegradability	Favorable biocompatibility with established biodegradation pathways	Variable biocompatibility depending on composition, size, and surface chemistry	Improved biological compatibility through polymer shielding of metallic components	[24-25,29,33-34]
Risk of systemic toxicity	Relatively low due to biodegradation and clearance mechanisms	Potential concerns related to long-term accumulation and oxidative stress	Reduced but still dependent on metallic content and biodistribution characteristics	[31,36,78]
Colloidal and physicochemical stability	Susceptible to aggregation and degradation under physiological conditions	High structural stability and resistance to degradation	Enhanced stability resulting from hybrid architecture	[25,27,50]
Theranostic functionality	Primarily therapeutic applications	Combined therapeutic and diagnostic applications with limited drug payload capacity	Integrated therapeutic delivery, imaging, and stimulus-responsive treatment capabilities	[20-23,57,67]
Manufacturing and scale-up feasibility	Established fabrication methods with moderate process complexity	Reproducible synthesis but strict control of physicochemical parameters required	Complex multistep fabrication and characterization processes	[50,74,76]
Regulatory and translational considerations	Relatively well-defined regulatory pathways	Regulatory concerns regarding long-term safety and biodistribution	Limited clinical experience and evolving regulatory frameworks	[50,73,76]
Current clinical development status	Multiple approved formulations and advanced clinical-stage candidates	Limited number of clinically translated systems	Predominantly preclinical and early translational development	[42-48]
Representative examples	PLGA nanoparticles, PEGylated nanoparticles, chitosan nanoparticles	Gold nanoparticles, silver nanoparticles, iron oxide nanoparticles	PLGA-gold hybrids, chitosan-gold hybrids, polymer-coated iron oxide nanoparticles	[20-31]

Despite encouraging preclinical outcomes, polymer-metal hybrid nanoparticle systems have not yet achieved successful clinical translation. One of the primary reasons is the complexity of their multifunctional design, which creates significant challenges in large-scale manufacturing, batch-to-batch reproducibility, quality control, and regulatory evaluation. Unlike conventional nanocarriers, hybrid systems integrate both polymeric and metallic components, resulting in more complicated pharmacokinetic, biodistribution, biodegradation, and toxicity profiles. Furthermore, insufficient long-term safety data, particularly regarding the accumulation and clearance of metallic nanoparticles, continue to hinder regulatory approval and clinical advancement.

Among the various hybrid nanoplatforms investigated, polymer-coated gold nanoparticles and iron oxide-based hybrid systems appear to be the most promising candidates for future clinical application. Gold nanoparticle hybrids have demonstrated considerable potential for targeted drug delivery, photothermal therapy, and imaging, while iron oxide-based systems offer additional advantages in magnetic targeting, hyperthermia, and diagnostic imaging. Polymeric carriers such as PLGA, PEG, and chitosan further enhance biocompatibility, drug-loading capacity, and controlled release characteristics, making these combinations attractive multifunctional therapeutic platforms.

The most challenging barriers to clinical implementation remain manufacturing scalability, reproducibility, regulatory standardization, and long-term safety assessment. Although advances in microfluidic synthesis, continuous manufacturing technologies, and precision nanomedicine are beginning to address some of these limitations, the absence of harmonized regulatory guidelines specifically designed for hybrid nanomaterials continues to slow their progression from laboratory research to clinical practice. Addressing these challenges will require interdisciplinary collaboration among materials scientists, oncologists, pharmaceutical developers, and regulatory agencies. Such collaboration will be essential to realize the full therapeutic potential of polymer-metal hybrid nanoparticle systems in breast cancer treatment.

Despite these challenges, several emerging technological advances may accelerate the clinical translation of polymer-metal hybrid nanoparticle systems in breast cancer therapy. Future advancements in polymer-metal hybrid nanoparticle systems are expected to be driven by the integration of artificial intelligence (AI)-assisted nanoparticle design, personalized nanomedicine approaches, and biomarker-guided targeting strategies to enhance therapeutic precision and patient-specific treatment outcomes. Furthermore, the combination of hybrid nanoplatforms with emerging immunotherapies may provide synergistic anticancer effects and improve treatment efficacy in resistant breast cancer subtypes. Advances in microfluidic and continuous manufacturing technologies are also anticipated to improve scalability, reproducibility, and quality control, thereby accelerating the clinical translation of next-generation nanomedicine platforms.

## Conclusions

Polymer-metal nanoparticle hybrid systems represent a promising and rapidly evolving strategy in breast cancer therapy, offering the potential to integrate targeted drug delivery, controlled release, and multimodal therapeutic functions within a single platform. By combining the biocompatibility and pharmacokinetic advantages of polymeric carriers with the optical, magnetic, and catalytic properties of metallic nanoparticles, these systems provide a rational approach to overcoming key limitations of conventional chemotherapy, including systemic toxicity, poor tumor selectivity, and multidrug resistance. The clinical success of approved nanomedicines such as Doxil and Abraxane confirms that nanotechnology can be successfully translated into oncology practice with measurable improvements in safety and therapeutic outcomes. However, despite encouraging preclinical evidence, polymer-metal hybrid nanoplatforms remain largely confined to experimental and early translational stages, with no established progression to late-phase clinical trials.

A major barrier to clinical implementation is the absence of standardized manufacturing protocols, limited long-term safety data, particularly for metallic components, and challenges in reproducibility and large-scale production. In addition, current regulatory frameworks established by the U.S. Food and Drug Administration and the European Medicines Agency, while comprehensive, are not yet fully adapted to address the complexity of multifunctional hybrid nanomaterials. Future progress in this field will depend on bridging the gap between nanomaterial engineering and clinical oncology through rigorous preclinical validation, harmonized regulatory guidelines, and well-designed clinical trials. Integration of clinically validated nanocarriers with hybrid systems may further accelerate translational potential. Overall, while significant challenges remain, polymer-metal nanoparticle hybrid platforms hold strong promise as next-generation precision nanomedicine tools for improving breast cancer treatment outcomes.

Despite the remaining scientific, manufacturing, and regulatory challenges, polymer-metal hybrid nanoparticle platforms hold considerable promise as next-generation precision nanomedicine tools for improving breast cancer treatment outcomes. The next decade will determine whether these multifunctional nanoplatforms evolve from promising laboratory innovations into clinically transformative technologies for precision breast cancer therapy. Although this narrative review highlights substantial progress in the field, further standardized preclinical investigations, well-designed clinical trials, and harmonized regulatory frameworks are essential to facilitate their successful clinical translation.
